# Tracking health system performance in times of crisis using routine health data: lessons learned from a multicountry consortium

**DOI:** 10.1186/s12961-022-00956-6

**Published:** 2023-01-31

**Authors:** Anne-Marie Turcotte-Tremblay, Borwornsom Leerapan, Patricia Akweongo, Freddie Amponsah, Amit Aryal, Daisuke Asai, John Koku Awoonor-Williams, Wondimu Ayele, Sebastian Bauhoff, Svetlana V. Doubova, Dominic Dormenyo Gadeka, Mahesh Dulal, Anna Gage, Georgiana Gordon-Strachan, Damen Haile-Mariam, Jean Paul Joseph, Phanuwich Kaewkamjornchai, Neena R. Kapoor, Solomon Kassahun Gelaw, Min Kyung Kim, Margaret E. Kruk, Shogo Kubota, Paula Margozzini, Suresh Mehata, Londiwe Mthethwa, Adiam Nega, Juhwan Oh, Soo Kyung Park, Alvaro Passi-Solar, Ricardo Enrique Perez Cuevas, Tarylee Reddy, Thanitsara Rittiphairoj, Jaime C. Sapag, Roody Thermidor, Boikhutso Tlou, Catherine Arsenault

**Affiliations:** 1 Department of Global Health and Population, Harvard T.H. Chan School of Public Health, 401 Park Drive, 3Rd Floor East, room L3-015A5, Landmark Center, Boston, MA 02215 USA; 2 Faculty of Medicine Ramathibodi Hospital, Mahidol University, Bangkok, Thailand; 3 School of Public Health, University of Ghana, Accra, Ghana; 4 Policy, Planning, Monitoring and Evaluation, Ghana Health Services, Accra, Ghana; 5 Swiss TPH, University of Basel, Basel, Switzerland; 6 World Health Organization, Vientiane, Lao People’s Democratic Republic; 7 School of Public Health, Addis Ababa University, Addis Ababa, Ethiopia; 8 Epidemiology and Health Services Research Unit CMN Siglo XXI, Mexican Institute of Social Security, Mexico City, Mexico; 9 Office of the Member of Federal Parliament Gagan Kumar Thapa, Kathmandu, Nepal; 10 Caribbean Institute for Health Research, University of West Indies, Kingston, Jamaica; 11 Hôpital Universitaire de Mirebalais, Zanmi Lasante, Arrondissement de Mirebalais, Haïti; 12 Faculty of Medicine Ramathibodi Hospital, Mahidol University, Bangkok, Thailand; 13 Ministry of Health of Ethiopia, Addis Ababa, Ethiopia; 14 Tufts Clinical and Translational Science Institute, Boston, USA; 15 Public Health Department, Faculty of Medicine, Pontificia Universidad Católica de Chile, Santiago, Chile; 16 Ministry of Health and Population, Government of Nepal, Kathmandu, Nepal; 17 School of Nursing and Public Health, University of KwaZulu-Natal, Durban, South Africa; 18 Korea National Health Insurance Services, Health Insurance Research Institute, Wonju, Gangwon-Do South Korea; 19 Division of Social Protection and Health, Inter-American Development Bank, Kingston, Jamaica; 20 Biostatistics Unit, South African Medical Research Council, Durban, South Africa; 21 Studies and Planning Unit, Ministry of Public Health and Population, Port-Au-Prince, Haiti; 22 Seoul National University College of Medicine, Seoul, South Korea; 23 Department of Global Health and Population, Harvard University, Boston, USA; 24 Université Laval, Québec, Canada

**Keywords:** Routine health information systems, Health systems, Quality of care, COVID-19

## Abstract

COVID-19 has prompted the use of readily available administrative data to track health system performance in times of crisis and to monitor disruptions in essential healthcare services. In this commentary we describe our experience working with these data and lessons learned across countries. Since April 2020, the Quality Evidence for Health System Transformation (QuEST) network has used administrative data and routine health information systems (RHIS) to assess health system performance during COVID-19 in Chile, Ethiopia, Ghana, Haiti, Lao People’s Democratic Republic, Mexico, Nepal, South Africa, Republic of Korea and Thailand. We compiled a large set of indicators related to common health conditions for the purpose of multicountry comparisons. The study compiled 73 indicators. A total of 43% of the indicators compiled pertained to reproductive, maternal, newborn and child health (RMNCH). Only 12% of the indicators were related to hypertension, diabetes or cancer care. We also found few indicators related to mental health services and outcomes within these data systems. Moreover, 72% of the indicators compiled were related to volume of services delivered, 18% to health outcomes and only 10% to the quality of processes of care. While several datasets were complete or near-complete censuses of all health facilities in the country, others excluded some facility types or population groups. In some countries, RHIS did not capture services delivered through non-visit or nonconventional care during COVID-19, such as telemedicine. We propose the following recommendations to improve the analysis of administrative and RHIS data to track health system performance in times of crisis: ensure the scope of health conditions covered is aligned with the burden of disease, increase the number of indicators related to quality of care and health outcomes; incorporate data on nonconventional care such as telehealth; continue improving data quality and expand reporting from private sector facilities; move towards collecting patient-level data through electronic health records to facilitate quality-of-care assessment and equity analyses; implement more resilient and standardized health information technologies; reduce delays and loosen restrictions for researchers to access the data; complement routine data with patient-reported data; and employ mixed methods to better understand the underlying causes of service disruptions.

## Introduction

The COVID-19 pandemic has prompted researchers and policy-makers around the world to use readily available routine health data to monitor the potential effects of the pandemic on essential health services and to make timely decisions [1]. Routine health information systems (RHIS) are tools used to record, store, retrieve and process a wide range of indicators routinely collected in the health system. RHIS are mainly intended to inform decision-making at the local and national levels [2]. While not primarily designed for research, RHIS can be useful to study service provision and utilization and the effects of public health interventions [3, 4]. Efforts continue towards improving these data systems. After analysing 133 country health information systems, WHO concluded that while some countries had built strong systems in specific areas, no country had a fully mature system capable of meeting their evolving needs for health information [5].

Following the COVID-19 pandemic, it is essential to consider whether health system performance in times of crisis can be tracked using routine health data. To date, however, the scientific literature has neglected this topic. A review of past studies using RHIS data found that over 50% of studies focused on maternal health and malaria, and 74.2% were conducted in sub-Saharan Africa. Thus, it is unclear whether RHIS databases can play a greater role in the scientific assessment of health system performance in diverse regions [3]. Moreover, Hoxha et al. [6] reviewed the technical, behavioural and organizational/environmental challenges that hinder the use of RHIS data in low- and middle-income countries (LMICs), but it is not clear how these issues unfold in times of crisis, such as a pandemic. To fill this gap, there is a need for knowledge on recent experiences and lessons learned across countries.

Since April 2020, the Quality Evidence for Health System Transformation (QuEST) network has used administrative and RHIS data to assess health system performance during COVID-19 in Chile, Ethiopia, Ghana, Haiti, Lao People’s Democratic Republic, Mexico, Nepal, South Africa, Republic of Korea and Thailand [7]. The initial aims of the collaboration were to (1) describe health system performance at the national and subnational levels over time and compare results across countries, and (2) estimate the effect of the COVID-19 pandemic and COVID-19-related policy responses on health services use, quality of care and institutional mortality from non-COVID-19 health conditions. Six out of 10 countries used an open-source, web-based platform called District Health Information Software 2 (DHIS2) as their RHIS [8]. DHIS2, the world’s largest RHIS platform, is used by 73 LMICs [9]. In the other four participating countries, various administrative datasets were used, including the Republic of Korea’s National Health Insurance Service (NHIS) Health Facility Claims Database, the National Health Database of the Ministry of Public Health of Thailand, and the information systems of the Ministry of Health of Chile (DEIS) [10] and the Mexican Institute for Social Security. We aimed to compile a large set of indicators representing the major health needs in participating countries. We focused on indicators available across multiple countries for the purpose of multicountry comparisons. Below we describe the resulting multicountry dataset and discuss the challenges and opportunities of working with these data to track health system performance during the COVID-19 pandemic.

## Assessing health system performance during COVID-19

Our multicountry collaboration has shown that administrative and RHIS data are a rich and important resource to track health system performance in times of crisis. Researchers using RHIS can access large databases remotely with relatively small budgets. In our study, RHIS data were used to describe changes in health services use and health outcomes at the regional and national levels during the pandemic. A multicountry analysis described the effects of the pandemic on 31 health services across 10 countries and discussed implications for pandemic preparedness [7]. In Mexico, an analysis estimated that 8.74 million patient visits were lost from April to December 2020 across nine types of health services [11] and supported the planning of a recovery strategy at the Mexican Institute for Social Security [12]. Similar analyses were conducted in Ethiopia and Nepal.

## Scope of health conditions

**Fig. 1:**
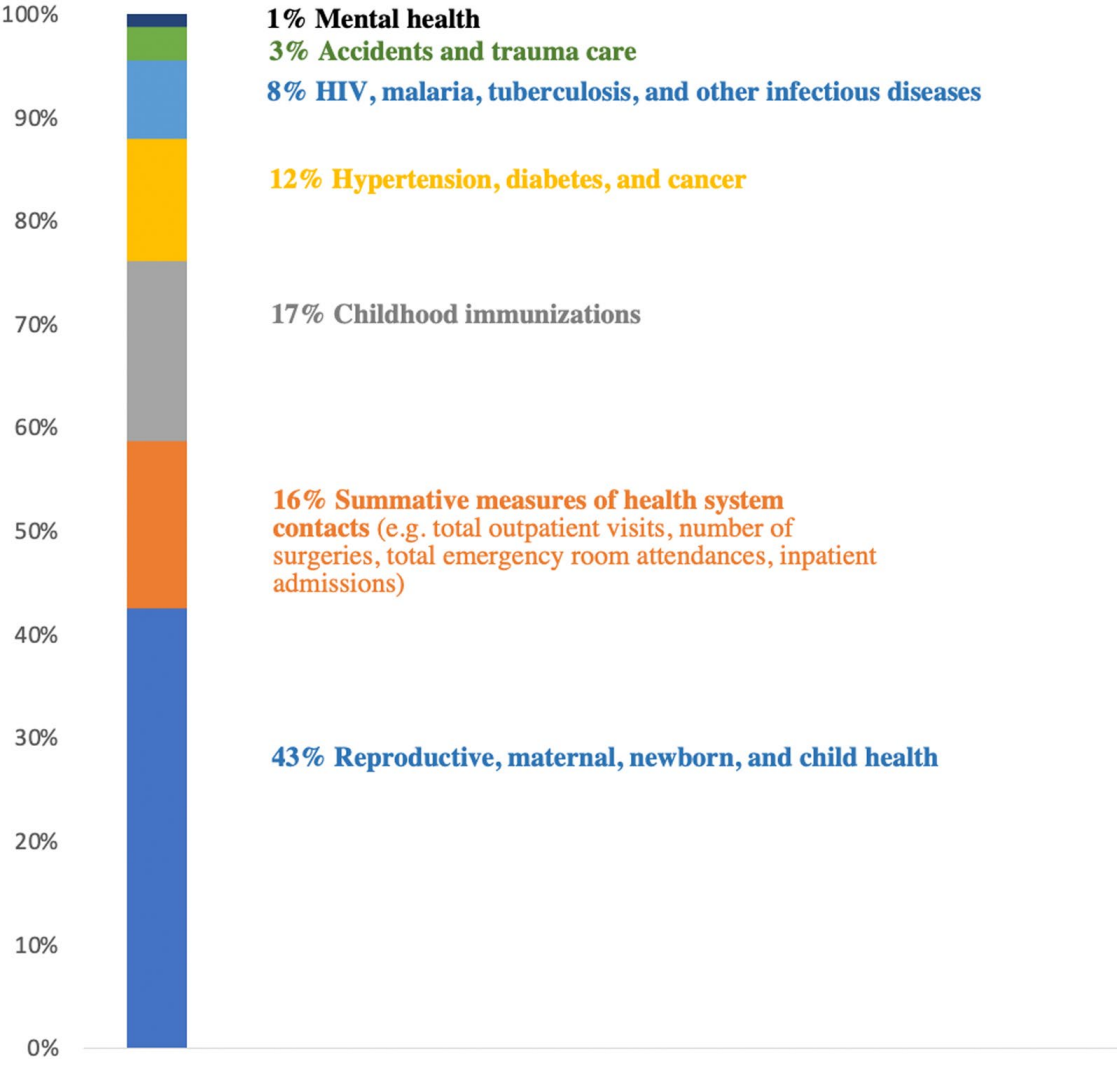
Scope of indicators compiled by health conditions (indicators were weighted by the number of countries that provided each of them)

Overall, the project compiled a total of 73 indicators. This represents the total number of indicators when adding up all the indicators collected in the 10 countries. The focus of some of these indicators overlapped while others did not. Each country provided between 13 and 39 indicators. For example, all 10 countries provided data regarding the number of deliveries that took place in health facilities. However, only three countries provided data related to mental healthcare. Figure 1 shows that 43% of the indicators pertained to reproductive, maternal, newborn and child health (RMNCH) (e.g. antenatal care, deliveries, family planning, stillbirths). Only 12% of the indicators were related to hypertension, diabetes or cancer care and outcomes. The predominance of RMNCH indicators does not represent the current global burden of disease, which in many countries has shifted towards noncommunicable diseases (NCDs) and injuries. For example, NCDs currently contribute to 71% of all deaths globally. Of these, 77% occur in LMICs [13]. The scope of health conditions covered in RHIS may be aligned with the priorities of development assistance donors [14].

We also found few indicators related to mental health services and outcomes within these data systems. Yet evidence suggests that the pandemic has led to an increased prevalence of major depressive disorders and anxiety disorders [15]. Given the high levels of economic losses and physical isolation during the pandemic, tracking and comparing mental health services and outcomes is crucial.

It should be noted, however, that we did not obtain the master list of indicators included in these data systems. The RHIS of some middle- and high-income countries contain more extensive indicators on chronic conditions, but these were not included in the multicountry dataset due to the lack of comparability with other countries. In the future, it would be interesting to repeat this analysis by obtaining the master list of indicators in each country to examine the comprehensiveness of available indicators [14].

## Data on quality of care

**Fig. 2:**
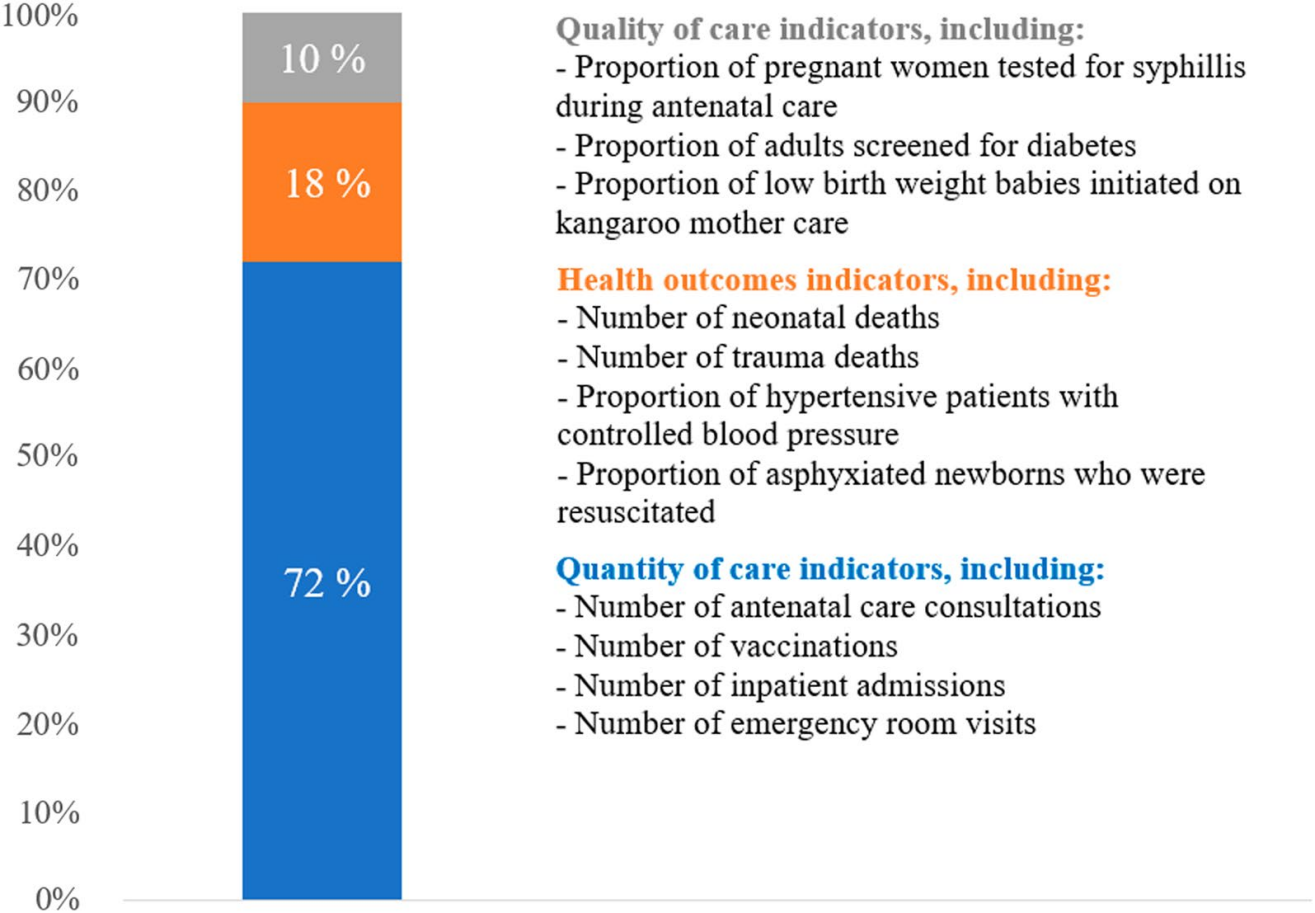
Scope of indicators compiled by indicator type (indicators were weighted by the number of countries that provided each of them)

Figure 2 highlights that about 72% of the indicators compiled were related to volume of services delivered (e.g. number of people receiving or using a specific health service), 18% to health outcomes (e.g. number of deaths, number of tuberculosis patients cured) and only 10% to the quality of care processes (e.g. appropriate testing, screening, treatments). A crisis may provoke changes in quality of care, such as in competence of care (i.e. systematic assessments, correct diagnoses, appropriate treatments, counselling, referral), as well as in system competence (i.e. safety, prevention and detection, continuity and integration, timely action, population health management) [16]. To facilitate the assessment of quality of care for a patient throughout the health system, RHIS should consider incorporating individual-level patient data, such as through electronic health records [17].

A crisis such as the COVID-19 pandemic can also affect user experience, which refers to the extent to which patients are treated with dignity and respect, are able to navigate the health system and have a health provider who communicates clearly and confidentially, without stigma or discriminatory behaviours [16]. Monitoring these dimensions of quality is crucial to assessing health system performance. These patient-reported experience measures must be measured through other types of data. For example, patient-reported data on their experience can be collected through household, phone or online surveys.

Quality-of-care indicators are also more informative if expressed as the proportion of patients who received a service (the numerator) among those eligible (the denominator). For example, the number of pregnant women tested for syphilis in a given quarter should be the numerator in proportion to the number of pregnant women who visited the facility that quarter, the denominator. Similarly, the number of women screened for breast cancer should be considered in proportion to the number of women who meet the age criteria for breast cancer screening. Estimates of the population in need of a service are sometimes inaccurate or outdated in RHIS [18, 19]. Moreover, a crisis can influence the proportions between numerators and estimated denominators. For example, border closures between neighbouring countries and the return of migrant workers can affect the population in need of services. Without accurate and appropriate denominators, it is more difficult to interpret RHIS data on health services.

## Population representativeness

Monitoring equity in access to healthcare services and health outcomes is also crucial in times of crisis. People needing care are not equally affected by pandemics and the mitigation measures implemented. RHIS databases enable researchers to examine regional inequities or differences between different types of facilities. However, analyses on the volumes of services and the quality of care received by particular socioeconomic groups are not always possible. It is important to track information on less advantaged populations and highest-risk populations (e.g. the elderly, patients with multimorbidity, people who are socially deprived). These variables could also be routinely collected and integrated into RHIS systems through the use of patient-level data. The fact that RHIS in some health facilities in LMICs is paper-based complicates the collection of these disaggregated data.

We found that some population groups and facility types were not represented in the databases of some countries. For example, Thailand’s Ministry of Public Health database only covers approximately 70% of the population [7, 20]. Many RHIS and administrative sources only include the public sector. Private hospitals and clinics and other private sector establishments where healthcare is provided (e.g. pharmacies, faith-based or nongovernmental facilities, prisons) are not always included. One reason for this absence is that private facilities are sometimes reluctant or have little incentive to report to RHIS. Thus, it is difficult to quantify compensatory shifts between different types of facilities occurring during a crisis (e.g. switching from public to private sector during the pandemic). The absence of complete datasets from all facilities nationwide not only matters in terms of inclusiveness (public vs private sector), but also can create potential selection biases. People using health services from different types of providers and/or with different funding mechanisms (public financing vs private insurance vs paying out of pocket) may have different health-seeking behaviours. In the future, public–private partnerships (shared information systems) in data management would facilitate comparisons between different types of facilities and make possible a more complete picture of health system shocks.

## Capturing non-visit or nonconventional care in RHIS

In times of health crisis, it is also important to capture services delivered in nonconventional ways. For example, in many countries (e.g. Chile, Mexico, Nepal, Republic of Korea, Thailand), we observed that strategies were developed to adapt health services to the pandemic, such as home delivery of drugs for chronic care patients and telephone outreach or telehealth consultations to replace in-person care in primary care facilities. Similarly, in South Africa, the Central Chronic Medicines Dispensing and Distribution (CCMDD) programme was intensified and expanded during this period, which allowed patients with chronic conditions to pick up their medication from more convenient locations, such as shopping malls, pharmacies and community halls. In Nepal, antiretroviral drugs were delivered door-to-door during the pandemic. However, some RHIS did not always capture these services in the database. For example, in Chile and Nepal, some information was kept on paper in nonsystematic formats and so was not analysed. As part of pandemic preparedness, information systems could be redesigned to permit healthcare workers to record services delivered in nonconventional ways in times of crisis. More broadly, this may also be an opportunity to better incorporate services delivered via different modalities, such as community outreach and telemedicine, which are currently underreported in RHIS and are likely to continue in the coming years.

## Data access

Stakeholders in this study faced issues related to data accessibility and ownership. Many RHIS are not currently treated as public goods. The National Health Database of the Ministry of Public Health of Thailand and the information systems of the DEIS were the only two datasets that were publicly available. Despite the fact that most RHIS systems do not contain personally identifiable information and that data are aggregated, governments sometimes set restrictions on access to stakeholders outside the ministries of health (e.g. researchers or citizens). Some restrictions may be set because of validation delays caused by the crisis. Chile, for example, offers open access for several indicators on the web, but there were some validation delays. In other countries, researchers required authorization from national health authorities to access the data. In our study, data access delays ranged from 3 months to 1 year. Once the preliminary analyses were conducted, the dissemination of results (e.g. policy briefs and dashboards) was delayed due to governments’ long approval processes. This limited the publication of live dashboards to share results publicly. Democratizing de-identified RHIS data to make them accessible to the general population, researchers, health system stakeholders and other actors who support health systems could facilitate their timely use, transparency, accountability and advocacy activities in times of crisis. This is especially important in certain LMICs where there are few mechanisms in place to demand health system accountability.

## Data quality

Ensuring the quality of RHIS data is an ongoing concern [3]. Across countries, those working with these data report recurring challenges, including frequent data updates due to delayed reporting by some facilities, difficulties differentiating between missing data and values of zero, indicator definitions being modified and indicators being removed. Researchers have explored strategies to deal with missing data in RHIS [21].

Another concern is whether the processes by which RHIS data are created are susceptible to shocks during times of crisis and whether this affects data quality. During the COVID-19 pandemic, for example, the monitoring and supervision of data processes may have been affected if limited resources were redirected. Also, healthcare workers may have had difficulty maintaining data entry while providing essential services. In some cases, data entry clerks may not have had full access to clinics or computers. However, in countries with disaggregated data, the pandemic did not appear to affect the quality of the indicators analysed [22]. Feedback mechanisms from subsequent administrative levels (district, regional, national, etc.) must also be in place to ensure data quality at the health facility level.

In countries with disaggregated data, our team conducted a series of verifications, cleaning and imputations before the data were analysed. This included imputing values of zero for missing mortality data if the service they related to had been provided (e.g. stillbirths and deliveries, inpatient deaths and inpatient admissions), limiting the datasets to facilities with stable reporting over time and removing positive outliers [7].

## Understanding the underlying causes of service disruptions

To identify the underlying causes of declines in healthcare, with a view to designing policy responses, RHIS data may need to be supplemented with other sources. For example, in Lao People’s Democratic Republic, the large declines in services around April 2020 may have been linked to the nationwide lockdown. This drop was likely caused by demand-side factors, but we do not know whether those were related to fear, restricted movement or other causes. Stakeholders needed to plan different measures depending on the causes. The RHIS data were not, in themselves, sufficient to identify the mechanisms by which different services were affected.

## Conclusion

Times of crisis require rapidly accessible information systems that can capture comprehensive portraits of health system performance, including quality of care. Our international collaboration has highlighted that administrative sources and RHIS can be useful to assess the effect of the COVID-19 pandemic and COVID-related policy responses on health services. These analyses led to the creation of live dashboards intended for timely sharing of data, a series of policy briefs distributed to governments, presentations to stakeholders and scientific publications. To continue improving the analysis of administrative and RHIS data in times of crisis, we offer the following recommendations:

- Ensure the scope of health conditions covered is aligned with the current burden of disease in each country (e.g. more indicators on mental healthcare, trauma and injuries are needed).
- Increase the number of indicators related to quality of care and health outcomes.
- Incorporate data on nonconventional care (e.g. telehealth and community drug deliveries).
- Continue improving data quality (including establishing standards for data review and data quality assessments), and expand reporting from private sector facilities.
- Move towards the collection of patient-level data through electronic health records to facilitate quality-of-care assessment and equity analyses.
- Implement more resilient and standardized health information technologies to be able to conduct regional, national and multicountry analyses.
- Reduce delays and loosen restrictions for researchers to access the data, in order to inform national responses in a timely manner.
- Complement routine data with patient-reported data (experience and outcomes) collected, for example, through phone or web surveys.
- Employ mixed methods to better understand the underlying causes of service disruptions and track health system performance.

The abovementioned challenges should be addressed to strengthen RHIS and guide decision-making in times of crisis. Improving RHIS should be considered a component of emergency preparedness.

## Data Availability

Not applicable.

## Abbreviations

CCMDD: Central Chronic Medicines Dispensing and Distribution
DEIS: Departamento de Estadísticas e Información en Salud del Ministerio de Salud de Chile
DHIS2: District Health Information Software 2
LMICs: Low- and middle-income countries
NHIS: National Health Insurance Service
NCD: Noncommunicable disease
QuEST: Quality Evidence for Health System Transformation
RMNCH: Reproductive, maternal, newborn and child health
RHIS: Routine health information system
